# Biomarkers Associated With *Leishmania infantum* Exposure, Infection, and Disease in Dogs

**DOI:** 10.3389/fcimb.2018.00302

**Published:** 2018-09-06

**Authors:** Carla Maia, Lenea Campino

**Affiliations:** Global Health and Tropical Medicine (GHTM), Instituto de Higiene e Medicina Tropical (IHMT), Universidade Nova de Lisboa (UNL), Lisbon, Portugal

**Keywords:** biomarkers, dog, exposure, infection, *Leishmania infantum*, leishmaniosis

## Abstract

Canine leishmaniosis (CanL) is a vector-borne disease caused by the protozoan *Leishmania* (*Leishmania*) *infantum* species [syn. *L*. (*L*.) *infantum chagasi* species in the Americas] which is transmitted by the bite of a female phlebotomine sand fly. This parasitosis is endemic and affect millions of dogs in Asia, the Americas and the Mediterranean basin. Domestic dogs are the main hosts and the main reservoir hosts for human zoonotic leishmaniosis. The outcome of infection is a consequence of intricate interactions between the protozoan and the immunological and genetic background of the host. Clinical manifestations can range from subclinical infection to very severe disease. Early detection of infected dogs, their close surveillance and treatment are essential to control the dissemination of the parasite among other dogs, being also a pivotal element for the control of human zoonotic leishmaniosis. Hence, the identification of biomarkers for the confirmation of *Leishmania* infection, disease and determination of an appropriate treatment would represent an important tool to assist clinicians in diagnosis, monitoring and in giving a realistic prognosis to subclinical infected and sick dogs. Here, we review the recent advances in the identification of *Leishmania infantum* biomarkers, focusing on those related to parasite exposure, susceptibility to infection and disease development. Markers related to the pathogenesis of the disease and to monitoring the evolution of leishmaniosis and treatment outcome are also summarized. Data emphasizes the complexity of parasite-host interactions and that a single biomarker cannot be used alone for CanL diagnosis or prognosis. Nevertheless, results are encouraging and future research to explore the potential clinical application of biomarkers is warranted.

## Canine leishmaniosis

Canine leishmaniosis (CanL) is a vector-borne zoonotic protozoan disease caused by *Leishmania* (*Leishmania*) *infantum* species [syn. *L*. (*L*.) *infantum chagasi* species in the Americas; (Mauricio, 2018)] which is transmitted by the bite of a female phlebotomine sand fly. CanL is endemic in approximately 50 countries and affects millions of dogs in Asia, the Americas, and the Mediterranean basin (WHO, 2010; Campino and Maia, 2018).

The outcome of infection in dogs is a consequence of intricate interactions between the protozoan *L. infantum* and the genetic background of the host (Baneth et al., 2008; Solano-Gallego et al., 2011; Campino and Maia, 2018). In addition, several non-genetic factors of the host, such as age, breed, gender, concomitant infections, immunological, and nutritional status as well as parasite virulence and previous exposure to *Leishmania* parasites can also affect infection outcome (Miró et al., 2008; Saridomichelakis, 2009; Hosein et al., 2017; Campino and Maia, 2018). The presence of *L. infantum* parasites in dogs can manifest as chronic infection without clinical signs lasting several years, self-limiting or severe illness that can rapidly progress to death (Solano-Gallego et al., 2009; Paltrinieri et al., 2010). In fact, not all dogs exposed to the parasite develop clinical signs and asymptomatic infections are much more frequent than clinical disease. On the other hand, a subclinical infection is not necessarily permanent and the break of parasite-host equilibrium can lead to the development of patent disease (Solano-Gallego et al., 2011). The progression of disease in susceptible dogs is characterized by an exacerbated humoral immune response, a depression of cellular immune response against the parasite, and the appearance of a panoply of clinical signs and/or physiopathological alterations. On the other hand, dogs considered resistant do not present clinical signs, have low levels of specific antibodies and low parasite levels, and present a robust cell-mediated immune response (Solano-Gallego et al., 2009; Paltrinieri et al., 2010; Maia and Campino, 2012; Hosein et al., 2017).

The commonest clinical signs in dogs with CanL are atrophic myositis of masticatory muscles, cutaneous alterations, lymphadenomegaly, onychogryphosis, and lesions derived from immune-complexes deposition such as glomerulonephritis, polyarthritis, or uveitis (Ciaramella et al., 1997; Maia and Campino, 2008; Paltrinieri et al., 2010; Solano-Gallego et al., 2011; Koutinas and Koutinas, 2014; Noli and Saridomichelakis, 2014; Meléndez-Lazo et al., 2018).

According to the Biomarkers Definition Working Group (2001), biomarkers are “biological parameters that can be objectively measured and evaluated as indicators of physiological or pathological processes, or a response to a therapeutic intervention,” and have been widely used in understanding several aspects of non-infectious and infectious diseases, such as exposure and susceptibility to infection (Carretón et al., 2014; Bryan, 2016). Despite clinical staging systems of CanL based on physiopathological abnormalities, clinical signs, serological alterations and/or direct detection of the parasite have been proposed (Solano-Gallego et al., 2009; Paltrinieri et al., 2010; Foglia Manzillo et al., 2013), the specific diagnosis of *L. infantum* is still a challenge (Maia and Campino, 2008). Apart from the confirmation of disease, other reasons for attempting laboratory diagnosis are the confirmation of *Leishmania* infection (in epidemiological surveys, to prevent infected dogs to be blood donors or be imported to non-endemic countries), and the monitoring of the response to treatment (Solano-Gallego et al., 2017; Campino and Maia, 2018). Therefore, the identification of reliable predictors for each purpose would represent an important tool to assist clinicians in follow-up monitoring, in determining an appropriate treatment and in giving a realistic prognosis to apparently healthy and sick dogs.

Here, we review recent research regarding the identification of biomarkers associated with *L. infantum* exposure, infection and disease in dogs and biomarkers useful to follow-up CanL and treatment efficacy.

## Biomarkers of exposure to *Leishmania infantum* or *L*. (*L*.) *infantum chagasi* vectors

*Leishmania* protozoa are transmitted by the bites of infected phlebotomine sand flies; thus, as the insect takes a blood meal its saliva is injected into the vertebrate host. Sand fly salivary glands secrete a complex array of active compounds that facilitate blood feeding and modulate host immune response having important consequences on the establishment or abrogation of infection (Lestinova et al., 2017). In addition, several components present in phlebotomine sand fly saliva are immunogenic to vertebrate hosts leading to development of saliva-reactive antibodies (Collin et al., 2009; Teixeira et al., 2010; Martín-Martín et al., 2014). A positive correlation between the level of specific antibodies to *Phlebotomus perniciosus* and *Lutzomyia longipalpis* saliva and the number of phlebotomine sand flies blood-fed have experimentally been demonstrated (Hostomska et al., 2008; Vlkova et al., 2011). Data from dogs living in endemic areas of leishmaniosis suggest the use of antibody response to saliva compounds as epidemiological biomarkers for monitoring vector exposure (Teixeira et al., 2010; Solcà et al., 2016; Kostalova et al., 2017; Quinnell et al., 2018). Moreover, the intensity of vector-exposure can be monitored throughout phlebotomine sand fly season as the host anti-saliva antibody response rapidly decreases in canine sera within 1 week after the last *P*. *perniciosus* exposure (Vlkova et al., 2011). Nevertheless, whether the antigenic response to phlebotomine sand fly saliva, which is developed whether the sand fly is infected or not, could be used as a risk marker for *Leishmania* infection in dogs remains controversial, as positive (Kostalova et al., 2015, 2017), negative (Vlkova et al., 2011) or no (Kostalova et al., 2017) correlation between the levels of anti-*P. perniciosus* saliva and antibodies against the parasite have been reported. Similar results were obtained with *L. longipalpis*; while Solcà et al. (2016) observed an increase in the number of dogs displaying antibodies to *L. longipalpis* saliva along with high parasite load, and therefore with disease severity, in the work performed by Quinnell et al. (2018) no association was observed between exposure to sand fly bites and disease progression. One reason for these contradictory results could be the fact that, with the exception of the work performed by Kostalova et al. (2017), anti-saliva antibodies have been detected using the whole content of the salivary glands reducing the specificity of detection due to a higher likelihood of cross-reactivity with saliva components from other sympatric non-vector phlebotomine sand fly species (Andrade and Teixeira, 2012; Lestinova et al., 2017).

## Genetic biomarkers of susceptibility to *Leishmania infantum* infection and disease

Genetic markers can be the responsible for the phenotypic variance of susceptibility of dogs to *L. infantum* as they control both pro- and anti-inflammatory cytokines as well as the cellular immune response to the presence of the parasite (de Vaconcelos et al., 2017).

Mutations and polymorphisms of the natural resistance-associated macrophage protein 1, *NRAMP1* gene (synonym of the solute carrier family 11 member 1, *Slc11a1*) have been associated with susceptibility to disease (Altet et al., 2002; Sanchez-Robert et al., 2005, 2008). *Slc11a1* gene encodes an ion transporter protein involved in the control of multiplication of *Leishmania* amastigotes and in macrophage activation. A predisposition to CanL has been associated with the haplotype of T antigen epitope TAG-8-141 and with two single nucleotide polymorphisms (SNPs) (A4549G in intron 6 and C4859T in exon 8) located in the *Slc11a1* gene in Boxer breed (Sanchez-Robert et al., 2005) and in different dog breeds (Sanchez-Robert et al., 2008), respectively. However, no significant differences in the expression of *Slc11a1* gene between phenotypically resistant and susceptible dogs or between primary canine monocyte-derived macrophages from *Leishmania*-free dogs with higher or lower resistance to intracellular survival of the parasites were found in the studies performed by Bueno et al. (2009) and Turchetti et al. (2015). The presence of the beta chain allele of the dog leukocyte antigen DLA–DRB1^*^01502 has been associated with susceptibility to CanL (Quinnell et al., 2003b) while the detection of SNPs 3, 4, 7 and 8 in the canine β*-defensin-1* gene has been associated with susceptibility to *L. infantum* or *L*. (*L*.) *infantum chagasi* infection (da Silva et al., 2017).

A genome-wide analysis using a dataset of 115 infected and 104 sick Boxers was assessed, as it is believed that multiple loci are responsible for the progression of *Leishmania* infection to clinical disease (Quilez et al., 2012). More than 170,000 single SNPs distributed throughout the genome were identified and, according to the authors, the significant predictive value of this genomic information may predict with an accuracy of ~0.29 the resistant and susceptible phenotype. Utsunomiya et al., 2015) identified two candidate loci in chromosome 1 and 2 involved in *Leishmania* infection using a panel of 145,000 SNPs distributed throughout the canine genome of 48 mixed-breed dogs (20 animals with PCR and ELISA positive to *Leishmania* and 28 negative controls). Candidate marker on chromosome 1 is related with notch signaling, which is key for macrophage activity and for T cell cluster of differentiation 4 (CD4^+^), while the candidate marker of chromosome 2 is related with the expression of interleukin 2 (IL-2) and IL-15, two cytokines with a pivotal role in the control and resolution of *Leishmania* infection (Utsunomiya et al., 2015).

## Hematologic and biochemical biomarkers of CanL

In infected dogs without or with light localized clinical signs, in which *Leishmania* presence was confirmed through direct methods and which have negative or low-titer anti-*Leishmania* antibodies, hematological and biochemical parameters are not usually changed (Solano-Gallego et al., 2009; Paltrinieri et al., 2010). On the other hand, laboratorial abnormalities are common in dogs with CanL (Table 1).

**Table 1:** Diagnostic, prognostic, treatment and post-treatment monitoring laboratory markers of canine leishmaniosis.

**Laboratory markers for**				**References**
Diagnosis	Common findings	Hematocrit	Mild to moderate normocytic and normochromic non-regenerative anemia	Reis et al., 2006b; Paltrinieri et al., 2016
			Neutrophilia	Ciaramella et al., 1997; Koutinas et al., 1999; Reis et al., 2006a; Paltrinieri et al., 2010; Nicolato et al., 2013; Meléndez-Lazo et al., 2018
			Thrombocytopenia	Koutinas and Koutinas, 2014; Paltrinieri et al., 2016
		Proteinogram	Polyclonal α- β- and γ-hyperglobulinem	Ciaramella et al., 1997; Koutinas et al., 1999; Giunchetti et al., 2008b; Paltrinieri et al., 2016; Meléndez-Lazo et al., 2018
			Hyperproteinemia	
			Hypoalbuminemia	
			Decreased albumin/globulin ratio	
		Biochemical parameters	Elevation of hepatic enzymes	Ciaramella et al., 1997; Koutinas et al., 1999; Giunchetti et al., 2008b; Meléndez-Lazo et al., 2018
			Elevation of renal enzymes	
			Increase of acute phase proteins (e.g., C-reactive protein, ferritin, haptoglobin, serum amyloid A)	Martinez-Subiela et al., 2014; Paltrinieri et al., 2016
			Increase of skeletal muscle enzymes	Vamvakidis et al., 2000
		Urinalysis	Proteinuria	Solano-Gallego et al., 2009; Paltrinieri et al., 2010, 2016
			Renal azotemia	Paltrinieri et al., 2016
	Less common findings	Hematocrit	Macrocytic hypochromic regenerative anemi	Ciaramella et al., 1997; Koutinas et al., 1999; Reis et al., 2006a; Paltrinieri et al., 2010; Nicolato et al., 2013; Meléndez-Lazo et al., 2018
			Monocytosis, lymphopenia, eosinopenia, or leukopenia	
		Hemostasis parameters	Serum hyperviscosit	Ciaramella et al., 2005; Petanides et al., 2008; Paltrinieri et al., 2010
			Thrombocytopathy	
			Impaired secondary hemostasis	
			Fibrinolysis	
			Hyperfibrinogenemia	
			Increase in prothrombin and activated partial thromboplastin times	
		Inflammation markers	Reduced serum activity of inflammatory markers (e.g., adenosine deaminase, butyrylcholinesterase)	Tonin et al., 2016
			Increased activity of leptin, matrix metalloproteinases, paraoxonase-1	Melo et al., 2011; Di Loria et al., 2014; Martinez-Subiela et al., 2014
		Oxidative stress markers	Reduced antioxidant levels and increased levels of oxidants with enhanced lipid peroxidation	Heidarpour et al., 2012; Almeida et al., 2017
		Urinalysis	Renal azotemia	Meléndez-Lazo et al., 2018
Prognosis	Common findings in dogs with poor prognosis	Hematocrit	Lymphopenia	Geisweid et al., 2012
		Proteinogram	Hypoalbuminemia	Geisweid et al., 2012; Paltrinieri et al., 2016
			Hyperproteinemia	
		Urinalysis	Proteinuria (urinary protein creatinine ratio-UPC ≥ 0.5)	Zatelli et al., 2003
			Azotemia (which may be associated with systemic hypertension)	Baneth et al., 2008; Paltrinieri et al., 2016
	Less common findings	Increased activity of gamma-glutamyl transferase and N-acetyl-b-N-glucosaminidase (associated with tubular injury)		Paltrinieri et al., 2016
		Increased apoptosis and reduced oxidation status and reactivity of neutrophils		Gomez-Ochoa et al., 2010; Almeida et al., 2013a,b
Treatment and post-treatment monitoring	Proteinogram	Decrease in globulin concentrations	2–6 weeks following treatment with antimonials; 3 months following treatment with marbofloxacin	Rougier et al., 2012; Rossi et al., 2014; Paltrinieri et al., 2016
	Biochemical parameters	Decrease of acute phase proteins values	C-reactive protein and serum amyloid A start to decrease within 2 weeks and return to previous values 1 month after treatment with meglumine antimoniate	Martínez-Subiela et al., 2003; Rossi et al., 2014
			Haptoglobin and C-reactive protein after long-term treatment with allopurinol	Sasanelli et al., 2007
	Urinalysis	Decrease of proteinuria within 4–8 weeks after treatment with allopurinol and meglumine antimoniate		Pierantozzi et al., 2013
	Remarks:	Serum creatinine and proteinuria should be tested:	At the end of the treatment and then 1 year after treatment (dogs in IRIS stage 1)	IRIS Glomerular Disease Study Group et al., 2013; Roura et al., 2013
			At the end of the treatment and then every 6 months (dogs in IRIS stage 2)	
			Frequently during treatment and then every 3 months (dogs in IRIS stage 3)	
			Frequently during treatment and then every 6 weeks (dogs in IRIS stage 4)	
		Albumin/Globulin ratio will remain low in dogs with persistent glomerular damage and proteinuria		Paltrinieri et al., 2016
		Complete regression of electrophoretogram alterations only 3–4 months after treatment		Torres et al., 2011

### Diagnostic markers

In dogs with clinical leishmaniosis, mild to moderate normocytic and normochromic non-regenerative anemia, typical of a chronic inflammatory disease, is the most common hematological abnormality (Reis et al., 2006b; Paltrinieri et al., 2016) and may be caused by decreased erythropoiesis due to disorders in the erythroid bone marrow compartment, or a decreased eryhopoietin production due to chronic renal failure. Although not so common, if anemia is due to an increased hemolysis (demonstrated by a positive Coombs test), macrocytic hypochromic regenerative anemia can be present.

Neutrophilia is also common in dogs with CanL (Ciaramella et al., 1997; Koutinas et al., 1999; Reis et al., 2006a; Paltrinieri et al., 2010; Nicolato et al., 2013; Meléndez-Lazo et al., 2018), and may be due to the inflammatory response resulting from the presence of parasites in multiple organs (Torrecilha et al., 2016). *Leishmania infantum* infection causes oxidative stress [i.e., a disruption in the normal balance between the production of reactive oxygen species (ROS) and antioxidant defenses] of canine neutrophils. The release of ROS from phagocytes present in inflammatory sites, leads to the consumption of antioxidant compounds (Torrecilha et al., 2016), representing a mechanism use by the parasite to evade the immune system. In fact, dogs presenting clinical signs have reduced antioxidant levels and increased levels of oxidants with enhanced lipid peroxidation (Heidarpour et al., 2012; Almeida et al., 2017). Other less common changes observed in leukocyte populations include monocytosis, lymphopenia, eosinopenia, or leukopenia (Ciaramella et al., 1997; Koutinas et al., 1999; Reis et al., 2006a; Paltrinieri et al., 2010; Nicolato et al., 2013; Meléndez-Lazo et al., 2018). Thrombocytopenia is also a common finding in dogs with leishmaniosis (Koutinas and Koutinas, 2014; Paltrinieri et al., 2016). Other coagulation disorders such as serum hyperviscosity, thrombocytopathy, impaired secondary hemostasis and fibrinolysis, hyperfibrinogenemia, increase in prothrombin and activated partial thromboplastin times have also been documented (Ciaramella et al., 2005; Petanides et al., 2008; Paltrinieri et al., 2010).

Protein alterations such as serum polyclonal α- β- and γ-hyperglobulinemia, hyperproteinemia, hypoalbuminemia and decreased albumin/globulin (A/G) ratio have been associated with disease progression (Ciaramella et al., 1997; Koutinas et al., 1999; Giunchetti et al., 2008b; Meléndez-Lazo et al., 2018). In fact, hyperglobulinemia in CanL is harmful, via the production of autoantibodies (e.g., immune-mediated thrombocytopenia), and/or circulating immune complexes generated in profuse amounts (Koutinas and Koutinas, 2014).

Elevation of hepatic and renal biochemical parameters are also commonly associated with the progression of the disease (Ciaramella et al., 1997; Koutinas et al., 1999; Giunchetti et al., 2008b; Meléndez-Lazo et al., 2018). Increase of acute phase proteins-APPs (e.g., C-reactive protein-CRP, ferritin, haptoglobin, serum amyloid A) are also common laboratorial findings associated with CanL (Martinez-Subiela et al., 2014; Paltrinieri et al., 2016).

An increase of the activity of skeletal muscle enzymes (e.g., creatine kinase-CK, lactate dehydrogenase-LDH) has also been documented in diseased dogs (Vamvakidis et al., 2000). Reduced serum activity of some inflammatory markers such as adenosine deaminase and butyrylcholinesterase in infected dogs have recently been reported (Tonin et al., 2016). An increased expression or activity of other molecules, such as leptin (Di Loria et al., 2014), matrix metalloproteinases (Melo et al., 2011) or paraoxonase-1 (Martinez-Subiela et al., 2014), has also been reported in blood samples of dogs with CanL.

### Prognostic markers

The presence of lymphopenia in diseased dogs is a marker of poor prognosis (Geisweid et al., 2012). In addition, as disease progresses increased apoptosis and reduced oxidation status and reactivity of neutrophils occurs (Gomez-Ochoa et al., 2010; Almeida et al., 2013a,b), which seems to be a critical mechanism of CanL pathogenesis (Almeida et al., 2017).

Regarding protein alterations, the severity of clinical score has been correlated with an increase of total proteins and a decrease in albumin concentration and therefore, hypoalbuminemia and hyperproteinemia are negative prognosis markers of CanL (Geisweid et al., 2012; Paltrinieri et al., 2016).

Severe (Paltrinieri et al., 2010) and very severe (Solano-Gallego et al., 2009) CanL with renal involvement should be suspected in the presence of proteinuria (i.e., when urinary protein creatinine ratio-UPC is equal or higher than 0.5) or renal azotemia (International Renal Interest Society; http://www.iris-kidney.com/guidelines/index.html)^1^. Proteinuria without renal azotemia seems to be secondary to immune complexes deposition at the glomerular level (Zatelli et al., 2003). Azotemia only becomes evident in an advance stage of disease and may be associated with systemic hypertension (Baneth et al., 2008; Paltrinieri et al., 2016). However, and according to the results recently obtained by Meléndez-Lazo et al. (2018), azotemia is not a common finding in dogs, as this biochemical alteration was only present in 6% of the 51 diseased dogs. Gamma-glutamyl transferase (GGT) and N-acetyl-b-N-glucosaminidase are the most popular urinary enzymes to measure tubular injury, which may be present secondarily to glomerular damage (Paltrinieri et al., 2016).

### Treatment and post-treatment monitoring markers

A decrease in globulin concentrations is expected in response to leishmanial treatment, however, A/G ratio will remain low in dogs with persistent glomerular damage and proteinuria as the concentration of albumin will remain low. Serum protein electrophoresis is more advisable to assess the efficacy of treatment and should not be run before 1 month of treatment initiation. A progressive decrease of globulins start to become evident after 2–6 weeks following treatment with antimonials (Rossi et al., 2014; Paltrinieri et al., 2016), and within 3 months following treatment with marbofloxacin (Rougier et al., 2012). However, the complete regression of electrophoretogram alterations requires at least 3–4 months (Torres et al., 2011).

Monitoring the concentration of APPs 1–2 weeks after the first administration of drugs provides earlier information regarding the success of treatment when other clinicopathological parameters are still abnormal (Paltrinieri et al., 2016). CRP and serum amyloid A values start to decrease within 2 weeks after treatment with meglumine antimoniate and return to previous values around 1 month (Martínez-Subiela et al., 2003; Rossi et al., 2014). Long-term treatment with allopurinol also significantly decreased the values of haptoglobin and CRP (Sasanelli et al., 2007).

Proteinuria tends to decrease in 4–8 weeks after treatment with allopurinol and meglumine antimoniate (Pierantozzi et al., 2013), but restore of renal function after leishmanicidal treatment depends on the severity of renal damage at the time of diagnosis. According to IRIS Glomerular Disease Study Group et al. (2013) and Roura et al. (2013), “serum creatinine and proteinuria of dogs in IRIS stages 3 or 4 should be frequently tested during the treatment period, while those in IRIS stages 1 or 2 should be tested at the end of the first treatment cycle. Post-treatment evaluation of dogs in IRIS stage 1 should be done after one year, in IRIS stage 2 every 6 months, in IRIS stage 3 every 3 months and in IRIS stage 4 every 6 weeks.”

## Immunological biomarkers of susceptibility and resistance to CanL

The course of *L. infantum* infection in dogs is tied-up to complex interactions of the host innate and adaptive components of the immune response, which dictates the clearance or persistence and multiplication of the parasite. The innate immune response has an important role in protection against the parasite, besides instructing the development of long lasting adaptive response (Moreno and Alvar, 2002; Reis et al., 2010; Hosein et al., 2017). The ability of the host to control *Leishmania* infection requires a strong cellular immune response, associated with the activation of T helper (Th)-1 cells producing interferon-gamma (IFN-γ), tumor necrosis factor alpha (TNF-α), and IL-2. Th1 is responsible for the activation of macrophages and concomitant intracellular killing of the parasites (Barbiéri, 2006; Carrillo and Moreno, 2009; Reis et al., 2010; Maia and Campino, 2012; Hosein et al., 2017) while active disease is associated with a mixed Th1/Th2 response (Carrillo and Moreno, 2009). Nevertheless, the immune response to the parasite is organ/tissue-specific as in different organs Th1, Th2 or mixed Th1/Th2 immune responses were observed and correlated with the absence or presence of clinical signs and with local parasite load (Reis et al., 2009; Maia and Campino, 2012; Hosein et al., 2017).

### Bone marrow

The absence of a specific immune response against *Leishmania* in bone marrow cells has been suggested as the expression of IFN-γ and IL-2, IL-4, IL-12 of asymptomatic and symptomatic dogs was similar to those of healthy animals (Barbosa et al., 2011). Nevertheless, the progression of *Leishmania* infection has been mainly associated with a pro-inflammatory environment, characterized by an elevated expression of TNF-α and IFN-γ by bone marrow cells (Quinnell et al., 2001b; Foglia Manzillo et al., 2006; Rodríguez-Cortés et al., 2016) together with a significant positive correlation between IL-4 levels and disease severity (Quinnell et al., 2001b). The absence of clinical signs in experimentally infected dogs was related with the no detection of this cytokine, together with the expression of inducible nitric oxide synthetase (iNOS) and of pro-inflammatory (TNF-α) and regulatory/anti-inflammatory [transforming growth factor-beta (TGF-β) and IL-10] cytokines (Maia and Campino, 2012). As IL-12 is involved in the inflammatory process that activates macrophages and enhances their microbicidal activity, it is not surprisingly the significant increased of its expression by bone marrow cells following treatment of dogs with meglumine antimoniate and allopurinol (Barbosa et al., 2011). The expression of the major histocompatibility complex (MHCII^+^) by bone marrow monocytes was also significantly increased after treatment, probably reflecting a rise in the presentation of *Leishmania* antigens (Alexandre-Pires et al., 2010).

### Liver

According to Michelin et al. (2011), liver is the main cytokine-producing organ during infection as IL-4, IL-10 and TNF-α production from liver extracts was higher than in spleen extracts both in infected asymptomatic and symptomatic dogs. In the work performed by Correa et al. (2007) the production of IL-10 and TGF-β1 by liver cells of infected dogs was lower in those with clinical signs. Similar results were obtained in experimentally infected dogs, as these cytokines were expressed by the liver cells in addition to IFN-γ and iNOS (Maia and Campino, 2012). The authors suggested that a high level of parasitism might have been associated with the absence of TNF-α. In fact, the down regulation of IFN-γ, TNF-α, IL-10, IL-17 cytokines, and iNOS by hepatocytes with disease progression in naturally infected dogs was reported (Nascimento et al., 2015). According to Rodríguez-Cortés et al. (2016), the anti-inflammatory/regulatory immune response observed in the liver at 6 months after experimental infection might be responsible for the absence of clinical signs even in the presence of a high parasite load. The down regulation of IL-22 transcription in liver samples was significantly associated with clinical disease in experimental infected dogs (Hosein et al., 2015). Nascimento M. et al. (2013) observed that the impairment of the expression of chemokines ligands-CCL (CCL1, CCL17, CCL26) and chemokine receptors-CCR (CCR3, CCR4, CCR5, CCR6, and CCR8) by liver cells in animals with clinical signs might result in deficient leukocyte migration and concomitant hampering of the immune response and disease development. In the study recently performed by Rodrigues et al. (2017) it was shown that *L. infantum* interacts with Kupffer cells inducing an anergic state that promotes immune tolerance and parasite survival. The silence imposed by the parasite was reverted by the presence of meglumine antimoniate.

### Lymph node

In lymph nodes, a balance between the expression of pro-inflammatory and anti-inflammatory cytokines expression seems to determine parasite load and clinical presentation (Alves et al., 2009; Barbosa et al., 2011; Maia and Campino, 2012). A high expression of IL-2, IL-12 (Barbosa et al., 2011), TNF-α, and IFN-γ has been reported in the lymph nodes of naturally (Alves et al., 2009; de Vasconcelos et al., 2016) and experimentally (Maia and Campino, 2012) infected asymptomatic dogs. Contrarily, IL-10 and TGF-β expressions were significantly increased in dogs presenting clinical signs, suggesting a role of these cytokines in disease evolution (Alves et al., 2009; de Vasconcelos et al., 2016; Rodríguez-Cortés et al., 2016). These results diverge from the ones obtained by Barbosa et al. (2011) where the expression of IFN-γ and IL-2 was higher in the lymph nodes of symptomatic dogs. Disease progression has also be linked to down regulation of IL-17, IL-22, and forkhead box P3 protein (FoxP3) in the lymph nodes of experimental infected dogs (Hosein et al., 2015). An association of high levels of IL-6 in lymph nodes of sick dogs with disorganization of the corticomedullar region, suggest this cytokine as good marker of active disease (de Vasconcelos et al., 2016).

In popliteal lymph nodes from natural infected dogs, a significant increased number of cytotoxic T cells (CD8^+^ T cells), together with decreased level of the cluster of differentiation (CD) CD21^+^ B cells and upregulation of MHCII^+^ molecules was observed (Giunchetti et al., 2008a). The levels of MHCII+ cells in lymph node lymphocytes were also increased after treatment with allopurinol and meglumine antimoniate (Alexandre-Pires et al., 2010). The CD4 T lymphocytes (CD4^+)^ and CD8^+^ T cells and Foxp3^+^ regulatory T cells (Tregs) frequencies in mononuclear cells of cervical and mesenteric lymph nodes of naturally infected dogs have also been evaluated (Figueiredo et al., 2014). Infected dogs had a higher expression of CD4^+^ and Foxp3^+^ cells than that of controls, but no correlation of these molecules with parasite load was found. The expression of Foxp3^+^ and CD4^+^ T cells was significantly higher in mesenteric lymph nodes of both asymptomatic and symptomatic dogs, respectively. Alexandre-Pires et al. (2010) also observed an expansion of CD4^+^ T cells subpopulations in popliteal and retropharyngeal lymph nodes of both symptomatic and meglumine antimoniate plus allopurinol treated dogs. The expansion of this cell subpopulation seems to be essential for the development of an efficient local immune response to *Leishmania* infection. In addition, the frequency of CD8^+^ T cells was significantly lower in lymph nodes of treated dogs than in asymptomatic dogs, suggesting that these cells might contribute to the reduction of parasite load through cytotoxic mechanisms (Giunchetti et al., 2008a).

### Peripheral blood

Despite not being the tissue of election for the multiplication and persistence of the parasite, the spectrum of cytokines and phenotypic cell profiles in peripheral blood have exhaustively been evaluated in naturally and experimentally infected dogs. However, results are often discrepant due to the use of different parameters to stage disease in dogs (i.e., based on the presence/absence of clinical signs and/or hematological and biochemical alterations) and with the different sensitivity and specificity of methodologies to determine the presence of specific antibodies and/or of the parasite (Maia and Campino, 2012).

IFN-γ expression/production by sera or by peripheral blood mononuclear cell (PBMC) lymphocytes non-stimulated or stimulated with soluble *Leishmania* antigen (SLA) from naturally and experimentally infected dogs was correlated with resistance to disease, asymptomatic status or mild disease (Santos-Gomes et al., 2002; Manna et al., 2006; Carrillo et al., 2007; Aslan et al., 2016; Abbehusen et al., 2017; Montserrrat-Sangrà et al., 2018)$. The lack of its production was observed both in symptomatic infected dogs (Carrillo et al., 2007) and in sick dogs with strong humoral response, high parasitaemia, and severe clinical disease (Solano-Gallego et al., 2016; Martínez-Orellana et al., 2017). On the other hand, high levels of IFN-γ production and expression was detected in naturally and experimentally infected dogs classified as symptomatic, indicating that this cytokine does not seem to be a good marker of resistance as it was not enough to prevent disease (Travi et al., 2009; Cortese et al., 2013). However, tracking IFN-γ concentration could constitute an important prognostic tool for immune monitoring in CanL, as its concentration increases with long-term anti-*Leishmania* treatment with meglumine antimoniate and allopurinol (Martínez-Orellana et al., 2017). Contradictory results have also been observed regarding the detection of several other cytokines. For instance, in the work performed by Pinelli et al. (1999), IL-4 and IL-10 were only expressed by PBMC stimulated with concanavalin A of dogs with clinical signs, while in other studies (Manna et al., 2006; Carrillo et al., 2007) both cytokines were detected in asymptomatic and symptomatic dogs. In the same line of reason, increased IL-10 production by PBMC stimulated with SLA was pointed as predictive marker of canine infection evolution (Boggiatto et al., 2010) and with splenic parasite load (Aslan et al., 2016), however, in other studies this cytokine was not considered a marker of disease severity (Santos-Gomes et al., 2002; Solano-Gallego et al., 2016). Similarly, IL-6 and IL-18 cytokines seem to have no role on infection outcome (Pinelli et al., 1994; Manna et al., 2006; Carrillo et al., 2007; Aslan et al., 2016) or to be markers of active disease (Lima et al., 2007) or asymptomatic infection (Chamizo et al., 2005). IL-2 and TNF-α production by stimulated PBMC of symptomatic and control uninfected dogs was significantly lower when compared with those from infected dogs without clinical signs (Pinelli et al., 1994), while IL-12 stimulated the production of IFN-γ by PBMC from symptomatic dogs experimentally or naturally infected (Strauss-Ayali et al., 2005). IL-2 levels were negatively correlated with splenic parasite loads in experimentally infected dogs, while no correlation between IL-12 and the number of parasites in the spleen was observed (Aslan et al., 2016). Due to the role of Tregs in the suppression of host immunity against *Leishmania*, these cells have also been evaluated in the peripheral blood of dogs naturally infected (Cortese et al., 2013). Results revealed a reduced percentage of Tregs CD4^+^, CD3^+^and Foxp3^+^ subsets on both asymptomatic and symptomatic infected dogs in comparison with non-infected controls.

Analyses of circulating leukocyte subpopulations pointed out the involvement of CD8^+^ lymphocytes in resistance to CanL (Pinelli et al., 1995; Reis et al., 2006b; Coura-Vital et al., 2011; Cortese et al., 2013) as increased levels of these cells were found in dogs with low parasitism. Further studies revealed an increase of CD3^+^ lymphocytes in infected dogs (Miranda et al., 2007), of CD5^+^ in symptomatic dogs (Reis et al., 2006b) and of CD4^+^ cells in dogs with a low parasite load (Reis et al., 2006b; Guerra et al., 2009). On the contrary, symptomatic dogs with high levels of parasitism in bone marrow have a decrease in CD21^+^ B cells and CD14^+^ monocytes and low levels of CD4^+^ and CD8^+^ T cells (Reis et al., 2006b). Low levels of circulating CD4^+^ in naturally infected dogs have also been associated with a higher infectivity to phlebotomine sand flies (Guarga et al., 2000). CD4^+^/CD8^+^ lymphocyte ratio has been evaluated with the rationale that development of clinical disease is accompanied by a reduction of CD4^+^ T cells. In fact, there is a decrease of CD4^+^ counts in the peripheral blood of sick animals, which tends to return to normal values after treatment (Moreno et al., 1999; Papadogiannakis et al., 2010). However, in some studies it was found a similar number of CD4^+^ counts in healthy and in infected dogs with no correlation between the clinical status (Miranda et al., 2007) probably reflecting individual variability (Paltrinieri et al., 2016). Therefore, and according to Paltrinieri et al. (2016) “CD4^+^/CD8^+^ ratio seems to be more suitable for monitoring the post-treatment follow-up rather than initial staging of clinical suspected dogs.” Studies using the saponin enriched-Leishmune® vaccine as immunotherapy revealed a sustained or increased proportions of CD4^+^ and CD21^+^ B lymphocytes and an increase proportion of CD8^+^ T cells in the peripheral blood of naturally and experimentally infected dogs (Borja-Cabrera et al., 2004, 2010; Santos et al., 2007). According to the studies performed by Araújo et al. (2008, 2009) Leishmune® promotes an increase of CD8^+^ T-cells activation, and induces a selective pro-inflammatory pattern with the production of IFN-γ and NO by peripheral blood lymphocytes and monocytes, respectively.

The analysis of the expression of the MHCII^+^ in peripheral blood lymphocytes has also lead to divergent results: up-regulation of MHCII^+^ expression was observed in asymptomatic (Reis et al., 2006b) and symptomatic dogs (Alexandre-Pires et al., 2010). On the other hand, the expression of this molecule was decreased in sick dogs with high parasite density in bone marrow (Reis et al., 2006b). In a cross-sectional exploratory study disease severity was characterized by an increase of the chemokine (C-X-C motif) ligand 1 (CXCL1) and CCL2 serum levels (Solcà et al., 2016). As the recruitment of neutrophils and monocytes is made by CXCL1 and CCL2 respectively, the increase production of these chemokines was probably related with enhanced parasite density (Solcà et al., 2016).

### Skin

After the inoculation of *Leishmania* parasites into the skin via phlebotomine sand fly female bite, several cells are involved in the activation of the innate immune system, with dendritic cells and macrophages playing a main role (Papadogiannakis and Koutinas, 2015). A Th2-biased immune response, with an increased expression of IL-4 (Brachelente et al., 2005), IL-10, and TGF-β (Rodríguez-Cortés et al., 2016) or overproduction of IL-4, IL-13, and TNF-α (Papadogiannakis and Koutinas, 2015) was associated with a high parasite burden and clinical disease. An increased parasite load was also associated with up upregulation of IL-10 and TNF-α in the skin of infected dogs (Pereira-Fonseca et al., 2017). On the other hand, a mixed Th1/Th2 cytokine profile and low levels of GATA-3 and Foxp3^+^ transcription factors in asymptomatic dogs indicates that in the absence of clinical signs or in cases with low number of parasites in the skin, a mixed inflammatory/regulatory immune response may be crucial (Menezes-Souza et al., 2011).

Menezes-Souza et al. (2012) reported a positive association between the CCL2, CCL4, CCL5, CCL21, and CXCL8 expression by the skin cells of naturally infected dogs with high cutaneous parasitism, while CCL24 expression was negatively correlated with parasite load. The cellular immunophenotyping and skin parasitism in symptomatic dogs has also been investigated (Fondevila et al., 1997; Papadogiannakis et al., 2005). An effective local immune response is associated to the activation of epidermal Langerhans cells, to the infiltration of dermis by CD8^+^ cells, to the upregulation of MHCII^+^ on keratinocytes, and by the absence or presence of few parasites. In contrast, the immune response in skin of dogs with clinical disease is characterized by a high number of plasma cells outnumbering T lymphocytes in the dermal infiltrate and by a high parasite load (Papadogiannakis et al., 2005).

### Spleen

Splenic architecture disruption due to CanL is characterized by the disorganization of lymphoid tissue with eventual atrophy and loss of leukocyte diversity (Sanchez et al., 2004). As with other tissues, contradictory results regarding the expression or production of cytokines and chemokines by spleen cells have been reported. On one hand, a positive correlation between IL-10 expression by spleen cells and increased parasitism and progression of the disease was found (Lage et al., 2007; Nascimento P. et al., 2013). On the other hand, no significant changes in the expression/production of this cytokine were reported, regardless the parasite load or clinical status of the dogs (Correa et al., 2007; Strauss-Ayali et al., 2007; Silva et al., 2014; Rodríguez-Cortés et al., 2016). Increased expression of TNF-α and IFN-γ by spleen cells was associated with reduced *Leishmania* burden (Nascimento P. et al., 2013). However, correlation between parasite load and the production of TNF-α by spleen extracts of dogs naturally infected has been documented, and according to the authors, it may represent an important marker for infection evolution (Michelin et al., 2011). Further, a worst disease prognostic was reported in dogs with a high expression or production of IFN-γ and splenic parasitism (Lage et al., 2007). In experimentally infected dogs, this cytokine was only expressed by tissues with high parasitic load (Maia and Campino, 2012), reinforcing its relation with the increase of parasitism (Lage et al., 2007). Persistence of parasites in the spleen has also been associated with early elevation of IL-4 expression by spleen cells in the presence of high levels of IFN-γ (Strauss-Ayali et al., 2007). Further, disease progression has significantly been associated to down regulation of IL-22 in the spleen of experimental infected dogs (Hosein et al., 2015), and to the down regulation of IFN-γ, IL-10, IL-17A, and iNOS in naturally infected dogs (Nascimento et al., 2015). The production of TGF-β by Tregs in the spleen was also evaluated but no correlation was found between the percentage of spleen Tregs producing this cytokine and the parasite load (Silva et al., 2014). An impairment of both pro-inflammatory and anti-inflammatory cytokines induced by splenic architecture breakage due to parasite presence has recently been reported (Cavalcanti et al., 2015).

An increase of the expression levels of IP-10, MCP-1, MIP1-α, and RANTES by spleen cells was observed during the follow-up of dogs experimentally infected (Strauss-Ayali et al., 2007). The increase of the chemokines was suggested to be associated with an accumulation of monocytes attracted by MCP-1 and MIP1-α, and with CD4^+^ Th1 and CD8^+^ cells recruited by IP-10 (Strauss-Ayali et al., 2007). The levels of these chemokines as well of IFN-γ significantly decreased after dogs were treated with allopurinol (Strauss-Ayali et al., 2007). In infected dogs the splenic expression levels of CCL1, CCL3, CCL17, CCL20, CCL26, CXCLl9, CCR3, CCR34, CCR36, and CCR38 were found to be reduced relatively to the expression levels in uninfected animals (Nascimento M. et al., 2013). Animals with disorganized lymphoid tissue presented lower CXCL13 expression and compared to those with organized lymphoid tissue, and the expression of this chemokine was associated with a higher frequency of severe disease (Silva et al., 2012). On the other hand, an increased expression of CCL2, CCL5, and CXCL10 by spleen cells was reported in symptomatic dogs in comparison with infected animals not showing clinical signs (Nascimento M. et al., 2013), and of CXCL12 in diseased animals and in those with disruption of the white pulp (Silva-O'Hare et al., 2016). All these data reinforces that the impairment of cell migration and the induction of long-lived plasma cells favors parasite replication and progression of the disease.

CCL21 and CCL19 chemokines are expressed by endothelial venules in lymphoid cells and organs (Ato et al., 2002). The binding of these chemokines to the CCR7 receptor of mature dendritic cells (DC) allows the migration of cells from the marginal zone to the peri-arteriolar region of the spleen. In mice chronically infected with *Leishmania* (*L*.) *donovani* it was shown that cellular immunosuppression is mediated by failure of DC migration due to the decreased chemokine secretion by endothelium and to the reduced DCs CCR7 expression (Ato et al., 2002). The immunotherapy with DC overexpressing CCR7 efficiently controlled infection in the spleen of infected mice (Ato et al., 2002). Therefore, molecules that can prevent the inhibition of this receptor would be of great interest for the control of leishmaniosis. *L. donovani* nucleoside hydrolase NH36 and its C-terminal domain, the F3 peptide have recently proven to be prominent antigens in the generation of preventive immunity to visceral leishmaniosis (Nico et al., 2018). Both antigens were able to control parasite loads in the spleen and liver of mice vaccinated with both antigens and then challenged with *L*. (*L*.) *infantum chagasi*. The imunotherapy with F3 antigens prevented the migrating defect of DCs by restoring the expression of CCR7 receptors (Nico et al., 2018). The ability of NH36, the main antigen of Leishmune®, to prevent or control leishmaniosis in dogs and mice by restoring a Th1 response was already be proven (Aguilar-Be et al., 2005; Borja-Cabrera et al., 2012).

### Other tissues

Figueiredo et al. (2014) evaluated CD4^+^ and CD8^+^ T cells and Foxp3^+^ Tregs frequencies and cytokine expression in the mononuclear cells of the jejunum and colon of dogs naturally infected with *L*. (*L*.) *infantum chagasi*. Frequencies and expression of IL-10, IFN-γ, TGF-β, TNF-α, Foxp3^+^, CD4^+^, and CD8^+^ were higher in jejunum, while IL-4 expression was significantly higher in the colon. A positive correlation between CD4^+^ in the colon and between CD4^+^ Foxp3^+^ Tregs in jejunum and parasite load was found. Infected animals had reduced CD8^+^ expression in both intestinal compartments compared to controls.

In *L*. (*L*.) *infantum chagasi* infected dogs CD3^+^ T lymphocytes were found to be the major components of the inflammatory infiltrate at the choroid plexus and in the brain; according to the authors during the advanced stages of leishmaniosis leukocytes might participate in the pathogenesis of neurological disorders (Melo et al., 2009). In fact, glial reactivity in dogs with leishmaniosis was correlated with T lymphocyte infiltration of the brain (Melo and Machado, 2011). Further, up-regulation of CCL3, CCL4, and CCL5, coherent with T lymphocyte accumulation, was observed in the brain of infected dogs (Melo et al., 2015). Apparently, the presence of parasite's DNA rather itself is enough to promote the development of a local immune response as no correlation between the downregulation of expression of IL-10, IL-12p40, and TGF-β by brain cells or the upregulation of IL-1-β, IFN-γ, and TNF-α and parasitism was found (Melo et al., 2013). Nevertheless, Grano et al. (2018) have recently found in the brain of infected dogs a moderate negative correlation between the levels of IL-1β and TNF-α and the number of parasites (Grano et al., 2018). Evidence of blood-cerebrospinal fluid barrier breakdown with the passage of T lymphocytes from the blood to the brain during CanL has been reported and related with the origin and progression of the neurological disorders (Grano et al., 2016).

Despite the recent advances made on the biomarkers related to the pathogenesis of *Leishmania infection* in the different organs and tissues, due to the invasive sampling and to the limited access to the tools to evaluate the biological markers, most of them cannot be used in a laboratory setting. Nonetheless, and taking into account the results described above, the inclusion in the laboratory diagnosis of the evaluation of the cytokines and phenotypic cell profiles of non-invasive samples, such as peripheral blood or lymph node aspirates, would probably represent a step forward for the prognosis and for monitoring the response to treatment.

## Serological biomarkers to *Leishmania infantum* infection and disease

CanL is often associated with a specific non-protective humoral response (Alvar et al., 2004; Maia and Campino, 2008; Miró et al., 2008; Solano-Gallego et al., 2009; Paltrinieri et al., 2016). However, the presence of antibodies to *Leishmania* alone is not conclusive of *Leishmania* infection, as it may simply reflect exposure to the parasite (Campino and Maia, 2018). Further, the production of specific antibodies is low on initial and late phase of infection and in infected dogs without clinical signs. Conversely, uncontrolled parasite dissemination is associated with gradually increase of antibody titers over time (Oliva et al., 2006), which will be high when the disease is evident (Campino, 2002; Solano-Gallego et al., 2009; Paltrinieri et al., 2016). While a direct relationship between tissue parasite density, clinical status and antibody titres is proven (Reis et al., 2006c; Dos-Santos et al., 2008; de Almeida Leal et al., 2014; Proverbio et al., 2014) low-to-medium antibody titres may also be detected in symptomatic dogs (Solano-Gallego et al., 2009, 2011, 2017; Paltrinieri et al., 2016).

Anti-*Leishmania-*specific canine IgG subclasses have extensively been investigated (Pinelli et al., 1994; Leandro et al., 2001; Iniesta et al., 2002, 2005; Cardoso et al., 2007) as an attempt to correlate the type of Th response, the subclass level and the clinical outcome of infection (Baneth et al., 2008; Maia and Campino, 2008). The majority of studies have focused on IgG1 and IgG2 responses and tried to link them with Th2-like susceptibility and Th1-like protective responses, respectively (Desplazes et al., 1995; Nieto et al., 1999; Iniesta et al., 2005; Cardoso et al., 2007; Rodríguez-Cortés et al., 2007a). A significant correlation between IgG, IgA, IgM (Rodríguez-Cortés et al., 2007a,b), and IgE (Iniesta et al., 2005; Reis et al., 2006c) and clinical signs have been found. After the launch of Leishmune® vaccine, which induce a strong humoral immune response, de Oliveira Mendes et al. (2003) evaluated if the production of IgG1 and IgG2 subclasses was able to distinguish the vaccinated dogs from those naturally infected. An association of IgG1 response to natural infection and IgG2 to a humoral response subsequent to the Leishmnune® vaccination was found (de Oliveira Mendes et al., 2003). In addition, IgG1/IgG2 ≥ 1 was associated to the sera of infected animals that evolve toward the disease while IgG1/IgG2 ≤ 1 was associated to the sera response of vaccinated dogs. Due to the low specificity of the polyclonal antisera commercially available to detect IgG subclasses results were often contradictory (Day, 2007). Thus, monoclonal antibodies to canine IgGs have been tested during natural and experimental infection. Nevertheless, a stable increase in the production of the four subclasses was observed with no indication of a practical use (Quinnell et al., 2003a; Strauss-Ayali et al., 2007).

Various quantitative serological methods such as the indirect immunofluorescence assay (IFAT) and enzyme-linked immunosorbent assay (ELISA) or the qualitative rapid immunochromatographic tests (ICT) are available for CanL diagnosis (Maia and Campino, 2008; Paltrinieri et al., 2016). Due to its high sensitivity and specificity (near 100% for both) IFAT is considered the reference method for anti-*Leishmania* serology in dogs (Gradoni and Gramiccia, 2008; EFSA AHAW Panel (EFSA Panel on Animal Health and Welfare), 2015). Sensitivity and specificity of ELISA is also quite high, especially when recombinant proteins are used as antigen (Paltrinieri et al., 2016). ICT are very attractive due to their single-test format, ease of use and quick response time (Maia and Campino, 2008). However, they only provide a qualitative result (i.e., presence/absence of specific reactive spots/bands) and their sensitivity is variable (Maia and Campino, 2008; EFSA AHAW Panel (EFSA Panel on Animal Health and Welfare), 2015; Paltrinieri et al., 2016). Therefore, in case of a positive result, a quantitative serology to obtain a titer for follow-up monitoring should be performed. In addition, and given the moderate sensitivity of most of the ICT, a negative result obtained with these devices in a clinically suspect dog should be followed by a quantitative test. According to Solano-Gallego et al. (2009, 2017) and Paltrinieri et al. (2016), “quantitative results provide by IFAT and ELISA reflect the final antibody titer (the last 2-fold serial dilution of sample providing a positive result). For ELISA, optical density values converted based on a reference titered sample should also be used. A titer is considered high if it is 4-fold higher than the threshold value of the laboratory (Solano-Gallego et al., 2009, 2017; Paltrinieri et al., 2016). Similarly, 4-fold titer variations in sequential samples of the same dog should be expected with seroconversions.” In it important clinicians to be aware that sequential samples should always be analyzed by the same method and in the same laboratory (Paltrinieri et al., 2016). Furthermore, it should be referred that serological titers not always correlate with the severity of the clinical signs (Ferrer et al., 1995; Manna et al., 2015) although asymptomatic dogs usually have low titers (Paltrinieri et al., 2010).

According to Paltrinieri et al. (2016), “in case of successful treatment, a decrease in antibody titers may be expected over time reaching values consistent with simple exposure (<4-fold the threshold value of the laboratory),” as in dogs living in endemic areas a complete disappearance of anti-leishmanial antibodies is unlikely. However, serology is not a reliable parameter to monitor treatment efficacy in the short-term (Ferrer et al., 1995; Miró et al., 2009; Torres et al., 2011; Manna et al., 2015). Albeit a significant reduction in titers can be detected 1 month post-treatment, a distinguishable decrease of titers is normally observed 6 months after initiation of therapy (Torres et al., 2011; Paltrinieri et al., 2016). In clinical relapses, a rise in antibody titers is observed (Manna et al., 2015).

As mentioned before, the presence of low levels of specific antibodies does not necessarily indicate an active infection. Further, the clinical presentation might be due to other pathologies. In these cases leishmaniosis diagnosis needs to be confirmed by the presence of the parasite or its components (direct methods) such as cytology, histology, immunohistochemistry, PCR or real-time PCR (Maia and Campino, 2008; Solano-Gallego et al., 2009; Paltrinieri et al., 2016; Campino and Maia, 2018). In addition, the vaccines available to prevent CanL have puzzled serological diagnosis, as most of the widely used tests are not able to discriminate between naturally infected and vaccinated dogs (Moreno et al., 2014; Paltrinieri et al., 2016; Solano-Gallego et al., 2017). Serological cross-reactivity with antibodies against other *Leishmania* species and pathogens such as *Trypanosoma cruzi, Ehrlichia canis*, and *Leptospira interrogans* (Ferreira et al., 2007; Porrozzi et al., 2007) is possible with some tests, especially those based on whole parasite antigens (Maia and Campino, 2008; EFSA AHAW Panel (EFSA Panel on Animal Health and Welfare), 2015).

## Cellular biomarkers to *Leishmania infantum* infection and disease

It is well established that susceptibility or resistance to *Leishmania* infection is mediated by cellular immune responses and several tools have been used to evaluate their role on the immunology and immunopathology of CanL (Maia and Campino, 2008; Paltrinieri et al., 2010; Reis et al., 2010).

Parasite-specific cellular immunity can be assessed by the Montenegro or leishmanin skin test (LST), which induces a delayed-type hypersensitivity response in dogs. *Leishmania* antigen, which consists of a suspension of inactivated parasites, is intradermal inoculated. A positive reading consists of an induration of over 5 mm in diameter obtained 48–72 h after inoculation. LST is negative during active disease, while during subclinical infection, early stage of clinical disease or after successful treatment is positive (Pinelli et al., 1994; Cardoso et al., 1998; Solano-Gallego et al., 2001; Fernández-Bellón et al., 2005). A strong and long lasting cellular immune response against the parasite has also been observed in Leishmune® vaccinated dogs, as a positive DTH response was present in immunized animals up to 41 months after vaccination (da Silva et al., 2000; Borja-Cabrera et al., 2002, 2008).

*Ex vivo* tests to assess *Leishmania*-specific cell-mediated immunity include lymphocyte proliferation assay and assays measuring IFN-γ in circulating lymphocytes (such as IFN-γ cytophatic effect inhibition bioassay and IFN-γ release assay). Lymphocyte proliferation assay consists on the stimulation of PBMC with soluble *Leishmania* antigen (SLA) and a mitogen with non-stimulated cells representing the negative control. Cell proliferation is expressed as a stimulation index (SI), which is obtained by the ratios of stimulated cells to non-stimulated cells). SI ≥ 2 are considered positive (Cabral et al., 1992, 1998; Pinelli et al., 1994; Leandro et al., 2001; Quinnell et al., 2001a; Fernández-Pérez et al., 2003; Santos-Gomes et al., 2003; Fernández-Bellón et al., 2005). Asymptomatic and resistant dogs present a strong proliferative response to leishmanial antigens while susceptible and diseased animals fail to respond respectively, to SLA and to mitogen (Abranches et al., 1991; Pinelli et al., 1994; Rhalem et al., 1999; Quinnell et al., 2001a; Strauss-Ayali et al., 2005). Lymphoproliferation is restored after successful leishmanicidal treatment (Bourdoiseau et al., 1997; Moreno et al., 1999; Rhalem et al., 1999; Fernández-Pérez et al., 2003).

Assays measuring IFN-γ allow quantifying the level of stimulation of a specific Th1-polarity immune memory response to *Leishmania* antigen (Fernández-Bellón et al., 2005; Rodríguez-Cortés et al., 2007a; Moreno et al., 2014; Zribi et al., 2017). The IFN-γ cytophatic effect inhibition bioassay detects the production of IFN-γ by circulating lymphocytes in cultured supernatants incubated in the presence/absence of SLA followed by incubation with canine kidney cells. IFN-γ production is expressed as the ratio of the reciprocal of the maximum dilution that protects 50% of the cell monolayer against vesicular stomatitis virus of stimulated vs. non-stimulated cells; values ≥ 2 are considered positive. The evaluation of its usefulness is very limited (Fernández-Bellón et al., 2005; Rodríguez-Cortés et al., 2007a) due to its cumbersome nature and to the use of a virus included in list A of the OIE.

The IFN-γ release assay (IGRA) allows rapid screening of IFN-γ-secretion in whole blood challenged with SLA. It seems to be a useful tool to assess exposure to *Leishmania* as positive IGRA responses were seen in infected dogs without or with mild clinical signs and in dogs without or with low parasite load, whereas negative IGRAs were identified in dogs with the highest parasitism (Zribi et al., 2017). The lack of IFN-γ production in dogs with severe clinical disease and high number of parasites on blood has also been reported (Solano-Gallego et al., 2016; Martínez-Orellana et al., 2017).

Canine macrophage leishmanicidal assay is also used to evaluate cell-mediated immune response via nitric oxide (NO) analyses. NO production by macrophages is the principal effector molecule mediating intracellular killing of *Leishmania* amastigotes by apoptosis. Cytokines such as IFN-γ and TNF-α secreted by activated T cells have been found to induce nitric oxide synthase (iNOS) and NO production facilitating parasite control (Green et al., 1990; Wanasen and Soong, 2008). The role of NO against CanL has been demonstrated by inducing antileishmanial activity in macrophages via the L-arginine NO pathway (Vouldoukis et al., 1996). After successful antimonial therapy canine macrophages regained the ability to control the parasites via increased NO production (Vouldoukis et al., 1996). Further, canine macrophages activated by a supernatant contained IFN-γ, TNF-α, and IL-2 were able to increase NO production and anti-leishmanial activity (Pinelli et al., 2000). Similarly, canine macrophages infected *in vitro* by *L. infantum* were able to produce NO after stimulation with cytokine-enriched PBMC supernatants (Panaro et al., 1998) and after stimulation with IFN-γ and bacterial lipopolysaccharide were also able to express iNOS (Sisto et al., 2001). A correlation between high iNOS expression by *Leishmania* infected canine macrophages and a low intracellular amastigote burden has also been reported (Zafra et al., 2008). The ability of canine macrophages to kill parasites through NO production as a measurement of long-term protection of dogs against *Leishmania* infection and disease has also been evaluated. Panaro et al. (2008) observed that in the first months after *Leishmania* diagnosis, the levels of NO produced by *Leishmania*-infected macrophages were higher in symptomatic dogs than in those without clinical signs. The role played by NO in leishmanicidal activity has also been demonstrated in the context of vaccination studies showing that immunized dogs develop long-lasting Th1cell-mediated immune responses against *L. infantum* or *L*. (*L*.) *infantum chagasi*. NO enhanced the anti-leishmanial activity of macrophages alone or co-cultured with IFN-γ producing autologous lymphocytes (Panaro et al., 2001; Lemesre et al., 2005; Rodrigues et al., 2007; Moreno et al., 2012, 2014), and has also been shown to mediate apoptosis of intracellular amastigotes (Holzmuller et al., 2005).

A practical and standardized assay to evaluate cellular immunity to *Leishmania* infection in clinical settings should be of practical use to help monitoring CanL and treatment outcome (Maia and Campino, 2008).

## Conclusion

The identification of biological parameters that can be indicators of pathological processes related to *L. infantum* infection or disease, or a response to *Leishmania* treatment or vaccination would represent a major progress in the control of canine leishmaniosis. Data gathered from several studies have identified potential biomarkers but none of them provided a strong evidence of their practical applicability on diagnosis. In addition, no single marker seems sufficient to be a direct correlate of resistance or susceptibility to disease, as a complex network of regulatory and counter-regulatory interactions involving several cytokines, chemokines and cell populations are involved in mounting an effective immune response. Therefore, multiple laboratorial, immunological and parasite-specific biomarkers should be considered together to obtain a general view of *L. infantum* infection and disease outcome. The visceral tropism of the parasites makes sampling challenging, as well as owner compliance, especially when invasive procedures need to be repeated. Therefore, the identification of biomarkers obtained from non-invasive samples is warranted. Finally, the development of vaccines to prevent CanL represents an important step forward to control the disease but it has complicated its diagnosis, so, biomarkers able to discriminate naturally infected from vaccinated dogs are urgent.

## Author contributions

CM writing and editing original draft. LC editing and review original draft.

### Conflict of interest statement

The authors declare that the research was conducted in the absence of any commercial or financial relationships that could be construed as a potential conflict of interest.

## Footnotes

^1^International Renal Interest Society (IRIS): Guidelines for Staging Chronic Kidney Disease (CKD). Available online at: http://www.iris-kidney.com (Accessed March, 2018)
